# The first aurochs genome reveals the breeding history of British and European cattle

**DOI:** 10.1186/s13059-015-0793-z

**Published:** 2015-10-26

**Authors:** Ludovic Orlando

**Affiliations:** Centre for GeoGenetics, Natural History Museum of Denmark, University of Copenhagen, ØsterVoldgade 5-7, 1350K Copenhagen, Denmark; Université de Toulouse, University Paul Sabatier (UPS), Laboratoire AMIS, CNRS UMR 5288, 37 Allées Jules Guesde, 31000 Toulouse, France

## Abstract

The first genome sequence of the extinct European wild aurochs reveals the genetic foundation of native British and Irish landraces of cattle.

See related Research article: www.dx.doi.org/10.1186/s13059-015-0790-2

## The multiple origins of domestic cattle

The agricultural transition that accompanied the Neolithic Revolution represented a turning point in our evolutionary history. By domesticating plants and animals, humans no longer depended on foraging and hunting for their subsistence and could, for the first time, produce their own resources, sustaining larger sedentary populations, and ultimately civilizations [1].

The domestication of cattle from the now-extinct wild aurochs (*Bos primigenius*) was essential in this process. Cattle livestock provided us with meat, leather, milk and other dairy products, but also enabled the transportation of goods and people in carts, and considerably helped the cultivation of soils by pulling ploughs. The first archaeological evidence for wild aurochs domestication appears in the Anatolian Fertile Crescent around 10,500 years ago, where cattle breeding developed for the next two millennia, before reaching Greece and further spreading into Europe [2].

## The domestication of European taurine cattle

Eurasian aurochs are well-described in the fossil record, but also on cave paintings, engravings and illustrations. They were one of the largest herbivores in Europe, with the biggest bulls reaching shoulder heights of 160–180 centimeters and massive frontal horns of up to 80 centimeters. Whether early European farmers fully adopted Middle Eastern domestic cattle or incorporated local wild aurochs into their livestock remains contentious. In contrast to dogs, goats, horses, pigs and sheep, for which wild populations still exist today, the last *B. primigenius* specimen died in Poland in 1627, precluding direct comparisons between living wild and domestic gene pools. Luckily, the survival of DNA molecules in archaeological bones and teeth offers direct access to the genetic makeup of extinct European wild aurochs, and can thereby help track their possible contribution to the European farming economy.

The first comprehensive survey of cattle mitochondrial DNA (mtDNA) variation that included aurochs sequences was carried out in 2001, and as in any ancient DNA study at the time, consisted of only a few hundreds of nucleotides [3]. The four aurochs analyzed, excavated in Britain, all belonged to a divergent haplogroup, called P, virtually absent from modern European breeds (the so-called humpless taurine cattle). Further mtDNA analyses of aurochs from mainland Europe, and ancient domestic cattle in the Middle East and throughout Europe confirmed a unique origin of the European domestic taurine mtDNA lineage in the Fertile Crescent, from which the original captive stock, of perhaps not more than 80 female founders, later expanded through the Danubian and Mediterranean routes [4]. Genetic mtDNA contact between Europe and the Middle East was virtually lost from 7000 years ago [4], and most European breeds apparently developed in situ with no mitochondrial influence from local aurochs, except perhaps in Italy, Poland and Switzerland where *B. primigenius* mtDNA variants can be occasionally found in modern and/or ancient cattle [5].

With high-throughput DNA sequencing technologies and recent methodological developments in ancient DNA research, whole genomes from ancient individuals can now be reconstructed. This has provided a fantastic opportunity to advance our understanding of domestication processes by delivering the genome sequence of wild animals that lived before domestication. This was done for the first time for horses [6], where wild alleles from a now-extinct wild population could be tracked within contemporary domestic breeds, thus revealing massive restocking from the wild, and highlighting the genes that were preferentially selected during domestication. A recent publication in *Genome Biology* applies this approach to cattle and reports the first genome sequence of a 6700-year-old male aurochs, excavated in Derbyshire, England [7].

## The first aurochs genome

From a total of 3.37 billion Illumina sequence reads, Stephen Park and colleagues [7] could identify 417 million sequences aligning uniquely and with high confidence to the cattle reference genome [8]. This represents an average 6.23-fold coverage of the genome, covering about 89 % of nucleotide positions at least once, and about 2.1 million candidate single-nucleotide polymorphism (SNP) and insertion-deletion (indel) variants. The genetic dataset also enabled the reconstruction of a high-quality mitochondrial genome sequence, clustering within the typical P haplogroup, which is absent from modern British cattle breeds. This is in line with earlier findings, which originally supported the contention that no British aurochs contributed to the genetic makeup of modern British cattle. However, the aurochs nuclear genome revealed a substantial amount of admixture with the ancestors of British breeds.

The support for this admixture is twofold. First, using the same method that unveiled the presence of Neanderthal ancestry in the genome of non-African anatomically modern humans, the authors [7] found an excess of shared derived polymorphisms between the genome of the aurochs and those of British and Irish breeds, in contrast to those of breeds from mainland Europe. Second, ancestry graphs placed the aurochs outside the range of modern taurine cattle diversity, but indicated substantial gene flow into British and Irish cattle. Clearly, in contrast to the domestication model built on mtDNA, British and Irish herders appear to have successfully incorporated wild aurochs variation into their livestock.

The limited density of the comparative SNP panel used in these analyses (representing about 15,500 high-quality SNPs genome-wide) precluded an identification of the aurochs genes that survive within present-day British and Irish cattle. One interesting possibility is that wild restocking helped adapt domestic cattle to Britain and Ireland, favoring the spread of alleles that had been naturally selected in local aurochs populations. Such cases of adaptive introgression have now been demonstrated in anatomically modern humans in Europe and Tibet, who acquired alleles from archaic hominins that were already adapted to their local environments [9]. There is no doubt that the newly released aurochs genome and the massive genome initiative of the 1000 bull genomes project (http://www.1000bullgenomes.com/) will soon help investigate whether this was also the case for domestic European taurine cattle.

For now, the authors [7] used shallow genome sequencing of 81 modern humpless taurine and indicine (humped zebu cattle, independently domesticated in the Indus valley) cattle to scan for molecular signatures of positive selection in the genomes of modern European breeds. One of these scans focused on loci where the aurochs showed the indicine allele and modern European taurine cattle an alternative allele at, or close to, fixation. This revealed 263 loci within potentially regulatory and protein coding regions, including eight non-synonymous mutations. Interestingly, one of these affected *DGAT1*, a gene exhibiting a major quantitative trait nucleotide for milk traits, and known to have been intensively selected in dairy cattle populations. Functional clustering enrichment analyses showed that genes involved in neurobiology, immunity, growth and metabolism were over-represented in the 263 variant set, indicating these biological processes as key targets of aurochs domestication. Immune genes might, for example, have been essential in the arms race against rapidly evolving pathogens, including those of human origin, as close proximity between herders and livestock could now facilitate host-shifts.

## Perspectives

As it provides the first aurochs genome, the study from Park et al. [7] represents a milestone in cattle genomics. However, it reports the genome of a single British animal, which lived about 4000 years after aurochs were first domesticated in the Fertile Crescent. Complete genome sequencing of early Neolithic aurochs from this region will be crucial for unraveling the genetic foundation of cattle domestication. As DNA decays at faster rates in warm environments, characterizing such genomes might prove extremely difficult. However, we can be confident that ever more sensitive methods that are tailored to the extraction and manipulation of short DNA molecules [8] will contribute to making this a reality; such methods have already helped characterize whole mitochondrial and nuclear genomes from much older specimens. Surveying the genomic diversity of wild aurochs throughout their whole European range will be equally important, as mtDNA variation suggests some population differences between mainland Italy and the British Isles. Outside Europe, characterizing the genomic variation of African and Asian aurochs will also be useful for tracking the origins and genetic makeup of African taurine and Asian indicine cattle.

The data in hand [7] have revealed that British aurochs significantly contributed to the genetic makeup of modern British and Irish cattle. As such, they reveal local restocking from the wild as an important component of cattle domestication, and reject simplistic scenarios where domestic livestock were first created in unique domestication centers, then expanded, ultimately sweeping away local wild populations. This result is in line with recent findings in other domestic animals, such as horses**,** dogs and pigs. It opens intriguing questions about the genetic mechanisms enabling maintenance of domestic phenotypes in the presence of homogenizing gene flow from the wild. It has been recently proposed that recurrent selection by breeders at particular loci underlying domestic traits could create domestication genomic islands maintaining population differentiation [10]. Future work scanning whole genome panels of wild and domestic ancestors of cattle, dogs, horses and pigs will help identify such regions and, if any, their common features, thereby illuminating the core features of the genetic mechanisms underlying animal domestication.

There is little doubt that ancient genomes will have an important role in such analyses, as the gene pool of domestic animals has been significantly reshaped within the past 200 years because of intense selective breeding and/or cross-breeding admixture, which partly erased genomic signatures of earlier domestication. Ancient DNA methods provide a unique opportunity to bypass such limitations and to chart through space and time the whole history of genomic changes accompanying animal domestication. The comparison of selection signatures will also reveal which animal traits were preferentially targeted in past societies.

Animal domestication is, however, likely to not just have remodeled the sequence of the genome. Microbiomes, for example, might also have changed in relation with dietary shifts, which possibly affected important phenotypic traits, ranging from the physiological to the behavioral. As wild and domestic animals show subtle changes in brain gene expression networks, transcriptional changes are also likely to have been an early component of domestication. A full comprehension of animal domestication will, thus, require extensive metagenomic, transcriptomic and epigenomic analyses of domestic and wild animals, including across developmental stages and tissues. This will be facilitated by the identification of functional elements in the genomes of domesticated animals being undertaken by the FAANG consortium. Interestingly, ancient DNA will also contribute to such analyses, because new approaches can reveal genome-wide methylation and nucleosome maps [9], thereby providing important insights on ancient gene expression levels. It will also be useful because ancient microbiomes can be reconstructed from deep-sequencing data of coprolites and dental plaques.

Finally, it is important to note that Park and colleagues [7] identified living breeds maximizing local wild ancestry, some of which, such as the Kerry cattle, are presently endangered. Developing conservation plans aiming at preserving such cryptic genetic variation in the British and Irish traditional landraces, and designing breeding programs targeting genomic ancestry blocks of aurochs origins, might eventually provide an opportunity to reverse-engineer aurochs-like animals, even in the absence of a proper de-extinction technology.

## Abbreviations

SNP: Single-nucleotide polymorphism
mtDNA: Mitochondrial DNA

## References

[1] Vigne JD, BocquetAppel J-P, Bar-Yosef O (2008). Zooarchaeological aspects of the Neolithic diet transition in the Near East and Europe, and their putative relationships with the Neolithic Demographic Transition. The Neolithic Demographic Transition and its Consequences.

[2] Bradley DG, Magee DA, Zeder MA, Bradley DG, Emshwiller E, Smith DB (2006). Genetics and the origins of domestic cattle. Documenting domestication: new genetic and archaeological paradigms.

[3] Troy CS, MacHugh DE, Bailey JF, Magee DA, Loftus RT, Cunningham P (2001). Genetic evidence for Near-Eastern origins of European cattle. Nature.

[4] Scheu A, Powell A, Bollongino R, Vigne JD, Tresset A, Cakirlar C (2015). The genetic prehistory of domesticated cattle from their origin to the spread across Europe. BMC Genet.

[5] Schibler J, Elsner J, Schlumbaum A (2014). Incorporation of aurochs into a cattle herd in Neolithic Europe: single event or breeding?. Sci Rep.

[6] Schubert M, Jonsson H, Chang D, Der Sarkissian C, Ermini L, Ginolhac A (2014). Prehistoric genomes reveal the genetic foundation and cost of horse domestication. Proc Natl Acad Sci U S A.

[7] Park S, Magee D, McGettigan P, Teasdale M, Edwards C, Lohan AJ, et al. Genome sequencing of the extinct Eurasian wild aurochs, *Bos primigenius*, illuminates the phylogeography and evolution of cattle. Genome Biol. 2015; In press. 10.1186/s13059-015-0790-2 PMC4620651 26498365

[8] Orlando L, Gilbert MTP, Willerslev E (2015). Reconstructing ancient genomes and epigenomes. Nat Rev Genet.

[9] Racimo F, Sankararaman S, Nielsen R, Huerta-Sanchez E (2015). Evidence for archaic adaptive introgression in humans. Nat Rev Genet.

[10] Frantz LA, Schraiber JG, Madsen O, Megens HJ, Cagan A, Bosse M (2015). Evidence of long-term gene flow and selection during domestication from analyses of Eurasian wild and domestic pig genomes. Nat Genet.

